# Changes in Biomarkers of Non-Alcoholic Fatty Liver Disease (NAFLD) upon Access to Avocados in Hispanic/Latino Adults: Secondary Data Analysis of a Cluster Randomized Controlled Trial

**DOI:** 10.3390/nu14132744

**Published:** 2022-06-30

**Authors:** Lorena S. Pacheco, Ryan D. Bradley, Cheryl A. M. Anderson, Matthew A. Allison

**Affiliations:** 1 Department of Nutrition, Harvard T.H. Chan School of Public Health, 665 Huntington Avenue, Boston, MA 02115, USA; 2 The Herbert Wertheim School of Public Health and Human Longevity Science, The University of California San Diego, 9500 Gilman Drive, La Jolla, CA 92093, USA; rybradley@health.ucsd.edu (R.D.B.); c1anderson@health.ucsd.edu (C.A.M.A.); 3 School of Public Health, San Diego State University, Hardy Tower Room 119, 5500 Campanile Drive, San Diego, CA 92182, USA; 4 Department of Family Medicine, The School of Medicine, The University of California San Diego, 9500 Gilman Drive, La Jolla, CA 92093, USA; mallison@health.ucsd.edu

**Keywords:** *Persea americana*, non-alcoholic liver disease, liver enzymes, inflammation, oxidative stress

## Abstract

Non-alcoholic fatty liver disease (NAFLD) is a public health concern and Hispanic/Latinos are disproportionately affected. There is evidence for favorable effects of dietary intake of monounsaturated fatty acids (MUFA) on NAFLD, yet studies examining avocados as a source of MUFA on hepatic function have not been assessed. We investigated the effects of low (3) vs. high (14) avocado allotment on biomarkers of NAFLD, oxidative stress, and NAFLD fibrosis score in a sample of Hispanic/Latino adults. Primary outcomes include hepatic function biomarkers [gamma glutamyltransferase (GGT), high-sensitivity c-reactive protein (hsCRP), and NAFLD fibrosis score]. Unpaired, two-sided *t*-tests were used to assess mean differences between intervention groups at 6 months and analysis of covariance models were used to adjust for diet quality and change in avocado intake from baseline to 6 months. Multivariable linear regression models evaluated the baseline and post-intervention association between avocado allotment group and outcomes, adjusting for covariates and stratifying by prediabetes status. No statistically significant differences were observed between low and high avocado allotment groups in liver enzymes, GGT, hsCRP or NAFLD fibrosis score. Findings persisted after stratifying by prediabetes status. Varied intake of avocados resulted in no effects on biomarkers of NAFLD in healthy adults, free of severe chronic disease.

## 1. Introduction

Non-alcoholic fatty liver disease (NAFLD) is one of the leading causes of chronic liver disease in the United States (US) and the most common cause of abnormal liver functions tests [1]. Steatohepatitis and hepatic fibrosis, both features of NAFLD, are prognostic indicators of an increased risk for morbidity and mortality, including that from cardiovascular disease (CVD) [2,3]. Notably, the majority of deaths in individuals with NAFLD are cardiovascular events, followed by hepatocellular carcinoma and end-stage liver disease [4,5]. Therefore, NAFLD is a serious public health concern affecting almost one third of the US population [6,7].

There is a significant racial and ethnic disparity in the prevalence of NAFLD in the US, with a disproportionately higher burden in Hispanic/Latinos [8]. In this regard, Shaheen et al., showed that Mexican-Americans had the highest prevalence of severe NAFLD (46%) relative to the other racial/ethnic groups (other Hispanic 30%, non-Hispanic Black 23%, non-Hispanic White 32%, and other race 28%); yet, only male Mexican-Americans, but not females, had a higher likelihood of both moderate and severe NAFLD relative to non-Hispanic whites [8]. Moreover, in a recent systematic review and meta-analysis, Rich et al. [9] reported significant racial disparities in NAFLD prevalence and severity in the US, with the highest proportion in Hispanic/Latinos (22.9%, 95% CI 21.6–24.1%), compared to non-Hispanic Whites (14.4%, 95% CI 14.0–14.8%), among participants in population-based studies. Regarding severity, ten studies evaluated NASH (nonalcoholic steatohepatitis) among NAFLD patients, and the pooled NASH prevalence was 31.4% (95% CI 30.1–32.7%). However, NASH prevalence was highest in Hispanics (45.4%, 95% CI 40.7–50.2%) and intermediate in Whites (32.2%, 95% CI 30.7–33.7%) [9]. In addition, a recent epidemiologic study showed that the mortality rates for NAFLD-cirrhosis and hepatocellular carcinoma have increased in non-Hispanic Whites followed by Hispanics [10]. 

Furthermore, the findings of a recent prospective study of 400 patients reported the prevalence of NAFLD among adult Hispanic/Latinos to be 19.4% [11], which differs among the Hispanic/Latino sub-populations. Particularly, Hispanic/Latinos of Mexican origin have the highest prevalence of NAFLD (33%), followed by Hispanic/Latinos of Puerto Rican origin (18%), and Hispanic/Latinos of Dominican origin (16%) [12]. Additionally, Le et al. [7] analyzed 1999–2012 data from the National Health and Nutrition Examination Survey (NHANES) to determine NAFLD prevalence and risk factors using two noninvasive techniques: the United States fatty liver index (USFLI) to ascertain NAFLD and NAFLD fibrosis score (NFS) to ascertain advance fibrosis. They reported that among 6000 individuals, 30.0% had NAFLD and 10.3% of these had advanced fibrosis. Some of the risk factors associated with NAFLD were male sex, slightly older in age (mean ± standard deviation, 53.2 ± 16.6 years among those with NAFLD vs. 47.3 ± 16.9 years among those free of NAFLD), Mexican-American ethnicity, lower income, and a lower education level. Similarly, and compared to their non-NAFLD counterparts, those with NAFLD were more likely to have cardiometabolic conditions such as metabolic syndrome, diabetes, ischemic heart disease, congestive heart failure, and a history of stroke, as well as a higher body mass index (BMI) and waist circumference. Conversely, in those with NAFLD, male sex and Mexican-American ethnicity were shown to be protective factors against advanced fibrosis [7]. 

Recent evidence suggests adherence to a dietary pattern high in monounsaturated fatty acids (MUFAs) is significantly associated with improved NAFLD-related markers including liver enzyme levels, liver fat content, fatty liver index, and steatosis [13]. MUFAs have been shown to enhance lipid oxidation and hinder lipogenesis, lowering hepatic steatosis [14]. Furthermore, other suggested favorable effects of MUFAs on NAFLD are associated with improved blood lipid profiles while stimulating removal of adverse circulating triglycerides [15,16]. However, trials thus far have been limited to examining the effects of olive oil consumption as main source of MUFAs [17,18,19], and have not investigated other foods sources. Therefore, examination of the impact of other MUFA-rich foods on hepatic health is warranted.

Avocados are a nutrient-dense food, high in MUFAs, and a rich source of antioxidants and polyphenolic compounds [20]. Although the effects of avocado on blood lipids have been previously studied [21], less is known about its effects on hepatic health. In this respect, there has only been one randomized controlled trial that has examined the impact of avocado consumption on CVD-related biomarkers of oxidative stress [22], which showed that a moderate-fat diet with 1 avocado/day for 5 weeks decreased circulating oxidized low-density lipoprotein (oxLDL) by 8.8% in adults with overweight and obesity, compared to baseline, after a run-in with the average American diet. Of note, none of the diets significantly affected plasma F2-isoprostane; a biomarker specific to lipid peroxidation. While the aforementioned study is the first to assess the effects of avocado on specific CVD-related oxidative stress measures, the effects of avocado on NAFLD biomarkers in healthy individuals free of chronic disease has not been reported.

In secondary analysis of data from the *Effects of Different Allotments of Avocados on the Nutritional Status of Families: A Cluster Randomized Controlled Trial* [23], we sought to determine the effects of high vs. low avocado intake on biomarkers of NAFLD and hepatic oxidative stress, with and without adjustment for adherence and other dietary adjustments. We hypothesized compared to low avocado intake, high avocado intake would have a beneficial effect on oxidative stress and hepatic health indicated by greater reductions in liver function tests and NAFLD fibrosis score.

## 2. Materials and Methods

### 2.1. Study Design and Population

*The Effects of Different Allotments of Avocados on the Nutritional Status of Families: A Cluster Randomized Controlled Trial* (i.e., the parent trial) was a cluster, randomized controlled trial of Hispanic/Latino families residing in San Diego County, California, and examined the impact of two levels of avocado allotment (i.e., 3 avocados/week/family vs. 14 avocados/week/family), plus a standard nutrition education intervention, on the nutritional status of Hispanic/Latino families. The primary outcomes of the parent trial included change in a family’s self-reported total energy and macro- and micronutrient intakes. 

Details on the methodology of the parent trial have been described elsewhere [23]. In brief, recruitment included a query search for potential participants of electronic medical records from a comprehensive health care system in South and Central San Diego County that primarily serves Hispanic/Latinos. Other recruitment strategies included telephone calls, flyers, and in-person contacts during clinic health fairs. A 14-day run period further evaluated and confirmed eligibility, as well as assessed commitment and adherence to study procedures by a study *promotora* (community health worker). Baseline measures were taken at this time, including a fasting blood draw at a local LabCorp clinic, and the head of household was self-identified by each family (one per family). After this, a computer-generated blocked, randomization sequence randomized the confirmed families.

**Figure 1:**
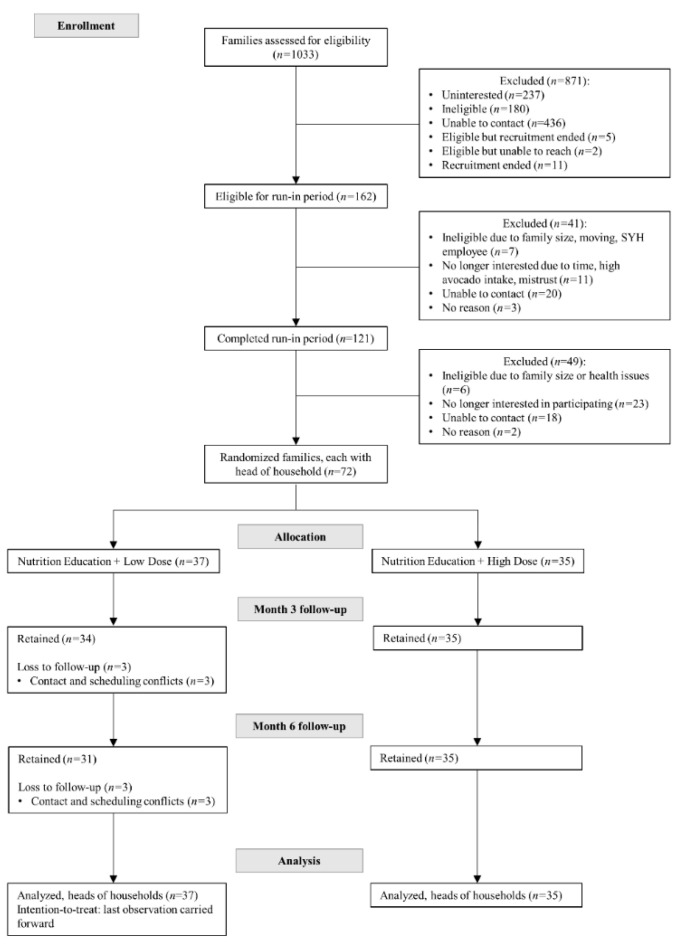
Consolidated standards of reporting trials (CONSORT) flow diagram of the Changes in Biomarkers of Non-Alcoholic Fatty Liver Disease (NAFLD) upon Access to Avocados in Hispanic/Latino Adults: Secondary Data Analysis of a Cluster Randomized Controlled Trial, ancillary study of the Effects of Avocado Intake on the Nutritional Status of Families Trial. This figure has been previously published in the parent trial [23].

Seventy-two families with at least 3 members of ≥5 years of age and residing in the same home, free of severe chronic disease, not on specific diets, and self-identified of Hispanic/Latino heritage, were randomized to one of two levels of avocado allotment (Low = 3/week/family or High = 14/week/family) for 6 months plus 12 bi-weekly standard nutrition education sessions led by *promotoras* (Figure 1 and Table 1). For each family randomized in the parent trial, a “head of household”, self-identified as the family member responsible for grocery shopping and meal preparation (i.e., in the Hispanic/Latino culture is commonly female, usually the mother, grandmother, or aunt). These heads of household completed all assessments and measurements in this secondary analysis. There were 71 women and 1 man who identified as the head of household.

The pre-planned ancillary study reported here was limited to head of household participants with complete hepatic function, inflammation, and oxidative stress biomarker values as part of the comprehensive metabolic panel of the original trial’s laboratory measurement at baseline and 6 months (Figure 1).

The Institutional Review Boards at the University of California San Diego and San Diego State University approved the original study protocol including subsequent analyses. Written, informed consent was provided by all participants. The original clinical trial was registered under clinicaltrials.gov study identifier NCT02903433.

### 2.2. Intervention

Heads of households and respective families in both intervention groups of the parent trial received nutrition education and avocados over a 6-month period. The nutrition education was identical for both groups and consisted of 12 bi-weekly culturally and language appropriate nutrition education standardized sessions derived from the United States Department of Agriculture MyPlate/MiPlato (http://www.choosemyplate.gov/ (accessed on 11 March 2016).) and aligned with the Dietary Guidelines for Americans [24]. These sessions were delivered by *promotoras* in participants’ homes with the goal of providing families with knowledge and tips to improve diet quality and meet nutritional goals, without individually counseling on energy restriction or elimination of any foods. Families were also provided with a recipe booklet to encourage inclusion of avocados in new ways. 

The dose of avocados in the low intake allocation group was 3 avocados/week/family, based on the average reported intake in a pilot study survey of selected individuals in the target population (data not shown). The rationale for this allotment was to standardize the control arm and reduce potential variability. The high intake allocation group was provided 14 avocados/week/family to allow for a substantial increase in daily intake (by allowing for up to 2 avocados/family/day). Avocados were delivered to families each week by the *promotoras* throughout the 6-month intervention period. Participating families were encouraged to not purchase additional avocados.

### 2.3. Avocado Intake and Intervention Adherence

Avocado intake was assessed using a validated [25], self-administered, web-based, VioScreen (VioCare, Inc., Princeton, NJ, USA) food frequency questionnaire (FFQ) [26] at baseline and 6 months. A continuous intake value was derived based on the number of avocados consumed by the head of household at 6 months since we are only examining the baseline and 6-month measurements. This variable was used as an adherence measure in the statistical analysis.

### 2.4. Hepatic Function, Inflammation, and Oxidative Stress

Heads of households completed a fasting blood draw at a local Laboratory Corporation of America Holdings (LabCorp, Burlington, North Carolina, USA) site at baseline and 6 months. This visit was scheduled within 5 days before or after their clinic visit (per the parent trial protocol). Research assistants helped coordinate the LabCorp appointment early in the morning after a 10–12-h overnight fast (no food or drink) for all study participants. In addition to a comprehensive metabolic panel measured as part of the clinical assessments in the parent trial, this ancillary study measured liver enzymes aspartate aminotransferase (AST), alanine aminotransferase (ALT), alkaline phosphatase; inflammation biomarker high sensitivity C-reactive protein (hsCRP); oxidative stress indicator gamma-glutamyl transferase (GGT); platelet count and serum albumin.

The NAFLD fibrosis score was calculated with the formula constructed and validated by Angulo et al., and includes age, AST, ALT, albumin, platelet count, BMI, and presence/absence of impaired fasting glucose or diabetes [27]. The score formula = −1.675 + 0.037 − age (years) + 0.094 − BMI (kg/m^2^) + 1.13 × impaired fasting glucose (IFG)/diabetes (yes = 1, no = 0) + 0.99 × AST/ALT ratio − 0.013 × platelet count (×109/l) − 0.66 × albumin (g/dl).

### 2.5. Covariate Assessment

Participant’s socio-demographic characteristics and lifestyle factors and behaviors were collected. Additional measures consisted of anthropometric measurements and physical activity. Anthropometric measurements included weight and height measured by a calibrated balance beam scale and stadiometer, respectively; and waist and hip circumference, which were measured with a semi-flexible tape measure, 2 cm above the iliac crest for waist and at the level of the widest circumference over the greater trochanters for the hip. Physical activity was measured via the global physical activity questionnaire (GPAQ) [28]. The dietary variables used in this study as covariates included total energy intake; intakes of fruits, vegetables and alcohol; and the healthy eating index (HEI)−2015, derived from the VioScreen FFQ [26].

### 2.6. Statistical Analysis

Descriptive statistics were used to characterize the study population by intervention group. Continuous variables were expressed as mean and standard deviation (SD), while categorical variables were expressed as frequencies and percentages. Normality was evaluated for all continuous variables. Unpaired, 2-sided *t*-tests were used to assess mean differences between intervention groups at 6 months for the biomarker outcomes: liver enzymes, GGT, hsCRP and NAFLD fibrosis score. These analyses were additionally adjusted for adherence to the HEI−2015 score in separate analysis of covariance (ANCOVA) models. ANCOVA models were also used to adjust for change in avocado intake from baseline to 6 months separately.

In multivariable analysis, four serial multivariable linear regression models evaluated the baseline and post-intervention association between avocado allotment group and biomarker outcomes adjusting for potential confounders, and mean between-group differences were determined. Model 1 adjusted for: age (years), moderate-vigorous physical activity (minutes/week), total energy intake (kcal/day), and alcohol intake (grams/day). Model 2 additionally adjusted for: change (from baseline) in fruit and vegetable intake (servings/day). Model 3 further adjusted for: change (from baseline) in waist circumference. Finally, model 4 adjusted for all variables in Model 3 as well as the diet quality score HEI−2015 at 6 months. 

All analyses were conducted in the full sample as well as in subgroups defined by prediabetes status, which was defined as having a fasting glucose of ≥100 mg/dL or hemoglobin A1c % of ≥5.7 or use of antidiabetic medications at baseline. Mean difference and standard error of the mean (SE) or 95% confidence intervals (CI) are presented, where appropriate. Primary analyses were conducted as intention-to-treat without adjustment for intervention adherence (avocado intake at month 6). We also performed per protocol adherence analysis which considered intervention adherence and limited to participants who completed the study. All *p* values presented are from 2-tailed analyses; *p* values of <0.05 were considered statistically significant. Analyses were conducted with SAS version 9.4 (SAS Institute Inc, Cary, NC, USA). 

## 3. Results

Of all heads of household trial participants, 83% were born in Mexico and, on average, had lived in the US for an average of 17.3 ± 12.6 years. The majority were married or cohabitating, homemakers, and their highest educational attainment was an Associate’s degree. Study participants had a mean age of 45.5 ± 9.9 years, a BMI of 30.5 ± 6.1 kg/m^2^. Less than a quarter of participants used medications, with 22.2% exclusively taking nonsteroidal anti-inflammatory drug (NSAIDs) (21.6% in the low avocado allotment group vs. 11.4% in the high avocado allotment group, *p*-value = 0.25). At baseline, the mean avocado intake in heads of household was 1.5 ± 1.4 and 1.5 ± 1.7 per week in the low and high avocado allotment groups, respectively. At 6 months, the mean intake of avocado was 1.7 ± 1.7 and 5.4 ± 3.3 per week in the low and high allotment groups, respectively. Blood pressure, lipid profile and other cardiometabolic risk measures are detailed in Table 1. Mean liver enzyme and GGT values were within normal range, and participants had a mean NAFLD fibrosis score of −2.2 ± 1.3. Changes in hepatic function measures at 6 months are shown in Table 2. Per intention-to-treat and per protocol analyses, and after adjusting for diet quality, there were no differences between low and high avocado allotment groups in liver enzymes, GGT, hsCRP or NAFLD fibrosis score. When stratifying by prediabetes status, participants with prediabetes randomized to the high avocado allotment study group showed a reduction in GGT at 6 months in per protocol analysis (Table 3). The mean 6-month change in GGT was +17.3 IU/L (95% CI: −2.1 to 36.8) for the low avocado allotment group and −10.3 IU/L (95% CI: −29.7 to 9.2) for the high avocado allotment group. The between-group mean difference was of borderline statistical significance (*p* = 0.06).

Changes in hepatic function outcomes at 6 months, after accounting for change in avocado intake, are shown in Table 4. We found no differences between low and high avocado allotment groups in hsCRP, GGT, liver enzymes, and NAFLD fibrosis score, per intention-to-treat or per protocol analyses. These results persisted after stratifying by prediabetes status (Table 5). As in the analyses not accounting for change in individual-level participant avocado intake, participants with prediabetes randomized to the high avocado allotment study group showed a reduction in GGT at 6 months in per protocol analysis. The mean 6-month change in GGT was +13.8 IU/L (95% CI: −5.3 to 32.7) for the low avocado allotment group and −6.6 IU/L (95% CI: −25.6 to 12.4) for the high avocado allotment group. However, the between-group mean difference did not reach statistical significance (*p* = 0.14).

The multivariable linear regression models assessing 6-month changes in hepatic function markers related to avocado allotment in all study participants, with and without stratification by prediabetes status, are shown in Table 6 and Table 7, respectively. After adjusting for intervention group, change in waist circumference and diet quality, there were reductions in GGT and alkaline phosphatase at 6 months. However, the associations did not differ (*p* > 0.05) (Table 6). In adjusted analyses, when stratifying by prediabetes status, participants without prediabetes had a −4.4 IU/L (95% CI: −11.5 to 2.6) and −5.6 (95% CI: −11.5 to 0.4) IU/L reduction in GGT and alkaline phosphatase, respectively, in per protocol analysis. These reductions did not differ (*p* > 0.05 for both) (Table 7).

## 4. Discussion

To our knowledge, this is the first study to test the effects of an avocado supplemented diet intervention on hepatic function and oxidative stress markers in healthy individuals, free of severe chronic disease. We found that Hispanic/Latino adults who were heads of households randomized to a high allotment of avocados (14/week/family) did not significantly reduce their levels of liver enzymes, GGT, hsCRP, or NAFLD fibrosis score, compared to adult heads of households randomized to a low allotment of avocados (3/week/family). Results denote little evidence of avocado effect on hepatic function and oxidative stress. However, there was a suggestive inverse association between high avocado intake and alkaline phosphatase and GGT among those heads of households with existing prediabetes, and evidence of a potential GGT-lowering effect in those with high avocado intake.

It is challenging to compare our findings with those of the existing evidence, since avocados have never been specific studied on biomarkers of NAFLD previously. However, a recent randomized controlled cross-over trial aimed to assess the effects of substituting carbohydrate energy in meals with half or a whole avocado on indices of metabolic and vascular health in overweight and obese middle-aged adults and measured similar biomarkers of inflammation and oxidative stress in the post-prandial period [29]. Although the cross-over trial examined different biomarkers than those used in our study, findings showed an increase in inflammatory marker interleukin−6 (IL−6) post-meal, but were not different between meals with/without avocado. Additionally, plasma concentrations of monocyte chemoattractant protein−1 (MCP−1), an adhesion molecule expressed by the stimulation of endothelial cells by CRP [30], did not differ between meals. Although we did not observe significant between group differences in fasting hsCRP after a longer-term intervention, we did not evaluate post-prandial effects. 

Elevated levels of liver enzymes ALT, AST, and GGT, are markers of NAFLD in both individuals with and without type 2 diabetes [31]. Our study population was generally healthy and free of NAFLD, and had normal values of these indicators at baseline. Thus, it is possible we did not observe changes in these hepatic health markers because they were too healthy. However, our results suggest avocado intake might be beneficial in those with existing metabolic abnormalities. In this respect, an in vivo animal study evaluated the effect of avocado oil, compared to olive oil, on hepatic function in sucrose-fed rats and found non-statistically significant differences in liver enzyme values between study groups [32]. While the nutritional profiles of avocado oil vs. whole avocado are not comparable, with the exception of MUFA content, the results are interesting. First, rats were separated into either a control group, which received a basal diet, or a sucrose-fed group, which received the basal diet plus 30% sucrose solution as drinking water. The animals had ad libitum access to food and water for 16 weeks before the sucrose-fed group was divided into one group of four groups: one group maintained a basal diet with sucrose and the three other groups were assigned to a sucrose plus 7.5% of either olive oil, avocado oil extracted via centrifugation, or avocado oil extracted via solvent, as primary source of dietary fat for 4 weeks. At the end of the study, levels of AST in both avocado oil groups (avocado oil extracted by centrifugation and extracted via solvent) were similarly reduced but not significantly different than either the basal diet with sucrose or the control group. However, the olive oil group showed significantly lower levels of AST, when compared with all other study groups. Additionally, the reductions observed in ALT and alkaline phosphatase did not differ when compared to all other study groups [32]. 

In clinical evaluations of high MUFA dietary patterns, studies that tested either the effects of a MUFA-rich diet in individuals with type 2 diabetes [17] or the impact of a Mediterranean-style diet in participants with NAFLD [18], demonstrated no significant reductions in ALT, AST, or GGT. Bozzeto et al. [17] assessed the 8-week effects of two widely recommended dietary approaches to manage type 2 diabetes (i.e., a high-carbohydrate/high-fiber/low-glycemic index diet and a high-MUFA diet) with and without the association of a structured physical activity program on liver biomarkers and liver fat. Although researchers reported no significant change in liver enzymes and HOMA-IR between groups, they observed a 29% significant decrease in hepatic fat content after 8-weeks with MUFA diet, independently of physical activity. This was a clinically significant decrease, suggesting that modifications in the intake of MUFA may positively impact fatty liver [17]. Similarly, Ryan et al. [18] examined the effect of the Mediterranean diet on steatosis and insulin sensitivity in twelve subjects free of diabetes (6 Females/6 Males), randomized to a cross-over 6-week dietary intervention study. All subjects followed both a Mediterranean diet and the control diet (low fat-high carbohydrate diet) with a 6-week wash-out period between each diet. While there were no significant reductions in liver enzyme levels, following the Mediterranean diet exhibited a 39 ± 4% significant relative reduction in liver fat content, measured by intrahepatic lipid, at 6-weeks. In contrast, when following the control diet only showed a 7 ± 2% relative decrease in liver fat content. However, there was significant improvement in insulin sensitivity following the MUFA-rich Mediterranean diet [18].

Though the mechanisms associated in the development of NAFLD remain unclear, a “multiple-hit” model has been proposed as an inclusive framework accounting for the interplay of multiple risk factors involved with the pathophysiology of NAFLD, including an individual’s dietary pattern, degree of insulin resistance, amount of visceral fat, state of inflammation, oxidative stress, the microbiome’s condition, and genetic susceptibility [33]. Oxidative stress, the relative overproduction of reactive oxygen species in excess of available antioxidant defenses, plays a key role as the starting point of the hepatic and extrahepatic damage. Subsequently, oxidative stress leads to lipid peroxidation and protein damage [34,35]. Free radicals react with unsaturated fatty acids (mainly poly-unsaturated fatty acids) producing lipid peroxides, which can damage endothelial tissue and induce impaired nitric oxide production and vasodilatory dysfunction [34]. 

Our findings were unexpected and contrary to our hypothesis since we would expect a beneficial effect on oxidative stress and hepatic in those participants randomized to the high avocado allotment group. As previously mentioned, a possible explanation for a lack of significant difference between avocado allotment groups could be that, although our population was overweight, they were healthy and without metabolic or severe chronic disease. Furthermore, small reductions were observed in those participants with prediabetes, particularly in the high allotment of avocado group, suggesting a potential floor effect for the participants overall, whereas there was some room for improvement in those with prediabetes. Additionally, it is conceivable that although the individual average weekly intake of avocado was estimated (at 6 months, 1.7 ± 1.7 vs. 5.4 ± 3.3 in low vs. high avocado allotment groups), the heads of household may not have consumed adequate avocados to have had an effect. Unfortunately, due to the design of the parent trial, we could not measure all hepatic function markers in all trial participants, which would have increased our sample size.

This study had several strengths including internal validity and the fact that this is the first clinical trial to assess risk of NAFLD indicators. Additionally, the study population consisted of Hispanic/Latino adult participants who were overweight/obese and thus at higher risk of developing NAFLD. This study also had limitations. First, we applied a fibrosis score to assess NAFLD status rather than a hepatic ultrasound, CT scan or gold standard biopsy. However, the NAFLD fibrosis score has been validated in 13 studies that included >3000 patients [36], and it is, at present, the most accurate non-invasive test for predicting advanced fibrosis for NAFLD in comparison studies [37,38], and has been endorsed by the American Association for the Study of Liver Diseases and the European Association for the Study of the Liver. Second, we were limited to collecting and reporting lab data on heads of households of families from the parent trial only, of whom the vast majority were women; thus, these results may not generalize to men or other trial participants. Although we would not anticipate sex differences, it would have been interesting to observe effects in both men and women had the sample allowed for sex-specific subgroups. Furthermore, our results cannot be extrapolated to the general population due to the nature of the study design. 

In future research, investigations on the effects of avocado on NAFLD should include individuals with insulin resistance and baseline elevated levels of GGT and/or established NAFLD to completely evaluate the effects of this food and its components.

## 5. Conclusions

After 6 months, adult heads of Hispanic/Latino households of families randomized to a low allotment of avocados (3/week/family) did not have significant changes in biomarkers of NAFLD, compared to adult heads Hispanic/Latino households of families randomized to a high allotment of avocados (14/week/family). These findings suggest increased access and intake of avocados had minimal effect on hepatic function and oxidative stress in healthy, overweight adults. Results should be interpreted with caution and future studies should consider avocado intake effects on individuals with cardiometabolic abnormalities. 

## Figures and Tables

**Table 1:** Baseline characteristics of randomized participants ^1^.

	Low Avocado Allotment (*n* = 37)	High Avocado Allotment (*n* = 35)
Characteristic		
Age, years	46.5 ± 11.3	44.5 ± 8.4
Female, %	97.3	100
Years lived in the United States	16.5 ± 12.6	17.7 ± 12.9
Country of birth, %		
Mexico	86.5	80.0
United States or Other	13.5	20.0
Heritage, %		
Central America	2.7	2.9
Mexico	97.3	97.1
Marital status, %		
Married or cohabitation	73.0	71.5
Separated, divorced, or widowed	18.9	20.1
Single	8.1	8.6
Highest level of education achieved, %		
High school	24.3	28.6
Trade school or Associate’s degree	23.6	31.4
Bachelor’s degree	18.2	17.1
Master’s degree or above	2.7	5.8
No diploma	10.8	8.6
Other	18.2	8.6
Employment status, %		
Employed for wages	24.3	28.6
Self-employed	10.8	8.6
Homemaker	48.7	40.0
Other	16.2	22.9
Total family income in US dollars/year, %		
Less than $30,000	40.5	54.3
Greater than $30,000	40.5	37.1
Unknow	18.9	8.6
Moderate vigorous physical activity, minutes/week	593.9 ± 672.9	582.9 ± 649.8
BMI, kg/m-squared	30.6 ± 6.1	30.5 ± 6.2
Waist circumference, cm	93.2 ± 13.4	95.0 ± 12.7
Systolic blood pressure, mmHg	116.9 ± 15.8	111.5 ± 15.7
Diastolic blood pressure, mmHg	73.4 ± 12.3	69.4 ± 8.8
Diabetes/prediabetes status ^2^, %	54.2	45.8
Glucose, mg/dL	102.3 ± 38.3	103.5 ± 30.4
Hemoglobin A1c%	5.9 ± 1.2	5.7 ± 1.0
hsCRP, mg/L	3.9 ± 4.5	2.9 ± 2.8
GGT, IU/L	25.1 ± 33.0	21.9 ± 15.0
AST, IU/L	21.0 ± 10.3	21.8 ± 12.0
ALT, IU/L	25.5 ± 21.8	23.3 ± 18.3
Alkaline phosphatase, IU/L	76.2 ± 21.8	71.3 ± 19.1
NAFLD fibrosis score ^3^	−2.2 ± 1.2	−2.1 ± 1.4

^1^ Mean ± SD (all such values). ^2^ Defined as a fasting glucose level ≥ 100 mg/dL or glycosylated hemoglobin ≥ 5.7% and/or reported use of glucose-lowering medication, at baseline. ^3^ <−1.455: predictor of absence of significant fibrosis (F0–F2 fibrosis), −1.455 to 0.676: indeterminate score, >0.676: predictor of presence of significant fibrosis (F3-F4 fibrosis). ALT, Alanine aminotransferase; AST, Aspartate aminotransferase; BMI, body mass index; GGT, gamma-glutamyl transferase; hsCRP, high-sensitivity c-reactive protein; NAFLD, Non-alcoholic fatty liver disease; US, United States.

**Table 2:** Changes in hepatic function outcomes at 6 months, per intention-to-treat and protocol adherence analyses.

Outcome	Within-Group Differences		Mean between-Group ^1^ Difference (95% CI)	*p*-Value ^2^
	Low Avocado Allotment	High Avocado Allotment		
	Mean (95% CI)	Mean (95% CI)		
	(*n* = 37)	(*n* = 35)		
hsCRP, mg/L				
Intention-to-treat	−0.2 (−0.9 to 0.5)	−0.2 (−0.6 to 0.3)	0.0 (−0.9 to 0.8)	0.95
Intention-to-treat ^3^	−0.3 (−0.8 to 0.3)	−0.1 (−0.7 to 0.5)	−0.1 (−1.0 to 0.7)	0.78
Protocol adherence ^4,5^	0.0 (−0.8 to 0.7)	−0.4 (−1.1 to 0.3)	0.4 (−0.7 to 1.4)	0.53
Protocol adherence ^3–5^	−0.1 (−0.8 to 0.7)	−0.3 (−1.0 to 0.4)	0.2 (−0.9 to 1.3)	0.70
GGT, IU/L				
Intention-to-treat	3.5 (−7.3 to 14.2)	0.0 (−3.8 to 3.7)	3.5 (−7.8 to 14.8)	0.54
Intention-to-treat ^3^	2.9 (−5.2 to 11.0)	0.5 (−7.8 to 8.9)	2.4 (−9.4 to 14.2)	0.68
Protocol adherence ^4,5^	7.1 (−2.9 to 17.1)	−2.7 (−12.0 to 6.6)	9.8 (−4.9 to 24.5)	0.19
Protocol adherence ^3–5^	6.4 (−3.7 to 16.6)	−2.1 (−11.5 to 7.4)	8.5 (−6.6 to 23.7)	0.27
AST, IU/L				
Intention-to-treat	−0.4 (−3.1 to 2.4)	−1.6 (−5.1 to 1.8)	1.3 (−3.0 to 5.6)	0.56
Intention-to-treat ^3^	−0.4 (−3.5 to 2.7)	−1.6 (−4.7 to 1.6)	1.2 (−3.3 to 5.6)	0.60
Protocol adherence ^4,5^	−0.3 (−4.1 to 3.5)	−1.7 (−5.3 to 1.8)	1.4 (−4.2 to 7.1)	0.61
Protocol adherence ^3–5^	−0.4 (−4.3 to 3.5)	−1.7 (−5.3 to 2.0)	1.3 (−4.5 to 7.1)	0.66
ALT, IU/L				
Intention-to-treat	−0.2 (−5.2 to 4.7)	−1.6 (−5.9 to 2.7)	1.4 (−5.1 to 7.8)	0.67
Intention-to-treat^3^	−0.3 (−4.9 to 4.2)	−1.5 (−6.2 to 3.2)	1.2 (−5.5 to 7.8)	0.73
Protocol adherence ^4,5^	0.1 (−5.6 to 5.8)	−2.0 (−7.3 to 3.3)	2.1 (−6.3 to 10.5)	0.62
Protocol adherence ^3–5^	0.0 (−5.8 to 5.8)	−1.9 (−7.3 to 3.6)	1.8 (−6.8 to 10.5)	0.67
Alkaline phosphatase, IU/L				
Intention-to-treat	−2.7 (−6.6 to 1.2)	−1.9 (−4.8 to 0.9)	−0.8 (−5.6 to 3.9)	0.73
Intention-to-treat ^3^	−2.8 (−6.2 to 0.6)	−1.8 (−5.3 to 1.7)	−1.0 (−5.9 to 4.0)	0.69
Protocol adherence ^4,5^	−2.2 (−6.4 to 2.0)	−2.8 (−6.7 to 1.1)	0.6 (−5.6 to 6.8)	0.85
Protocol adherence ^3–5^	−2.3 (−6.6 to 1.9)	−2.7 (−6.7 to 1.3)	0.4 (−6.0 to 6.8)	0.91
NAFLD fibrosis score				
Intention-to-treat	0.1 (−0.1 to 0.2)	0.2 (−0.1 to 0.4)	−0.1 (−0.4 to 0.2)	0.43
Intention-to-treat ^3^	0.1 (−0.1 to 0.2)	0.1 (−0.1 to 0.3)	−0.1 (−0.4 to 0.2)	0.51
Protocol adherence ^4,5^	0.1 (−0.1 to 0.3)	0.1 (−0.1 to 0.3)	0.0 (−0.3 to 0.3)	0.99
Protocol adherence ^3–5^	0.1 (−0.1 to 0.4)	0.1 (−0.1 to 0.3)	0.0 (−0.3 to 0.4)	0.91

^1^ Mean difference is low-high avocado allotment group. ^2^ From unpaired *t*-test or ANCOVA model (adjusted for Healthy Eating Index 2015 score at Month 6 and/or intervention adherence), where appropriate. ^3^ Adjusted for Healthy Eating Index 2015 score at Month 6. ^4^ Adjusted for intervention adherence. ^5^ Sample size by intervention group allocation (low avocado allotment/high avocado allotment): 31/35 at month 6. ALT, Alanine aminotransferase; AST, Aspartate aminotransferase; GGT, gamma-glutamyl transferase; hsCRP, high-sensitivity c-reactive protein; NAFLD, Non-alcoholic fatty liver disease.

**Table 3:** Changes in hepatic function outcomes at 6 months, stratified by prediabetes status ^1^, per intention-to-treat and protocol adherence analyses.

Outcome	Within-Group Differences				Mean between-Group Difference ^2^ (95% CI)			
	Low Avocado Allotment(*n* = 37)		High Avocado Allotment(*n* = 35)					
	Mean (95% CI)		Mean (95% CI)		Mean (95% CI)	*p*-Value ^3^	Mean (95% CI)	*p*-Value ^3^
	Prediabetes		Prediabetes		Prediabetes		Prediabetes	
	Yes (*n* = 18)	No (*n* = 19)	Yes (*n* = 15)	No (*n* = 20)	Yes		No	
hsCRP, mg/L								
Intention-to-treat	−0.2 (−1.7 to 1.2)	−0.2 (−0.7 to 0.4)	−0.2 (−1.1 to 0.7)	−0.2 (−0.7 to 0.3)	−0.0 (−1.7 to 1.6)	0.95	0.0 (−0.7 to 0.7)	0.99
Intention-to-treat + HEI ^4^	−0.3 (−1.4 to 0.8)	−0.1 (−0.7 to 0.4)	−0.1 (−1.4 to 1.1)	−0.2 (−0.8 to 0.3)	−0.2 (−1.9 to 1.5)	0.82	0.1 (−0.7 to 0.9)	0.77
Protocol adherence ^5–7^	−0.2 (−1.6 to 1.3)	0.1 (−0.6 to 0.8)	−0.3 (−1.8 to 1.1)	−0.4 (−1.0 to 0.2)	0.2 (−2.0 to 2.3)	0.86	0.5 (−0.4 to 1.5)	0.29
Protocol adherence + HEI ^4–7^	−0.2 (−1.6 to 1.2)	0.2 (−0.5 to 0.9)	−0.3 (−1.7 to 1.2)	−0.5 (−1.1 to 0.1)	−0.1 (−2.1 to 2.2)	0.98	0.7 (−0.3 to 1.7)	0.16
GGT, IU/L								
Intention-to-treat	8.1 (−15.0 to 31.1)	−0.9 (−2.5 to 0.7)	−2.6 (−8.0 to 2.8)	1.9 (−3.5 to 7.3)	10.7 (−12.8 to 34.1)	0.35	−2.8 (−8.3 to 2.8)	0.31
Intention-to-treat + HEI ^4^	7.4 (−9.5 to 24.2)	−0.4 (−4.5 to 3.7)	−1.8 (−20.3 to 16.7)	1.5 (−2.5 to 5.5)	9.1 (−16.0 to 34.3)	0.46	−1.9 (−7.8 to 4.0)	0.52
Protocol adherence ^5–7^	17.3 (−2.1 to 36.8)	−2.4 (−7.5 to 2.7)	−10.3 (−29.7 to 9.2)	3.0 (−1.5 to 7.5)	27.6 (−1.6 to 56.8)	0.06	−5.4 (−12.8 to 2.1)	0.16
Protocol adherence + HEI ^4–7^	16.5 (−3.0 to 35.9)	−1.8 (−7.1 to 3.6)	−9.4 (−28.8 to 10.0)	2.5 (−2.2 to 7.1)	25.9 (−3.4 to 55.1)	0.09	−4.2 (−12.2 to 3.8)	0.29
AST, IU/L								
Intention-to-treat	0.0 (−4.7 to 4.7)	−0.7 (−4.2 to 2.9)	−5.3 (−12.2 to 1.6)	1.1 (−2.1 to 4.3)	5.3 (−2.5 to 13.0)	0.18	−1.8 (−6.4 to 2.8)	0.44
Intention-to-treat + HEI ^4^	−0.3 (−5.5 to 4.9)	0.0 (−3.4 to 3.3)	−4.9 (−10.6 to 0.8)	0.5 (−2.7 to 3.7)	4.7 (−3.1 to 12.4)	0.23	−0.5 (−5.3 to 4.2)	0.82
Protocol adherence ^5–7^	−1.0 (−7.6 to 5.5)	−0.5 (−4.8 to 3.8)	−4.2 (−10.8 to 2.3)	0.8 (−2.9 to 4.6)	3.2 (−6.6 to 13.0)	0.51	−1.3 (−7.6 to 5.0)	0.67
Protocol adherence + HEI ^4–7^	−1.3 (−7.8 to 5.2)	0.6 (−3.8 to 4.9)	−3.9 (−10.4 to 2.6)	0.0 (−3.8 to 3.8)	2.6 (−7.2 to 12.4)	0.59	0.6 (−6.0 to 7.1)	0.86
ALT, IU/L								
Intention-to-treat	0.5 (−9.1 to 10.1)	−0.9 (−5.4 to 3.5)	−6.3 (−15.3 to 2.8)	1.9 (−1.4 to 5.2)	6.8 (−6.0 to 19.6)	0.29	−2.8 (−8.2 to 2.5)	0.28
Intention-to-treat + HEI^4^	0.2 (−8.5 to 8.8)	−0.3 (−4.2 to 3.6)	−5.9 (−15.4 to 3.7)	1.3 (−2.5 to 5.0)	6.0 (−6.9 to 19.0)	0.35	−1.6 (−7.1 to 3.9)	0.56
Protocol adherence ^5–7^	−0.4 (−11.2 to 10.5)	−0.5 (−5.5 to 4.5)	−5.3 (−16.2 to 5.6)	1.4 (−2.9 to 5.8)	5.0 (−11.4 to 21.3)	0.54	−1.9 (−9.2 to 5.4)	0.60
Protocol adherence + HEI ^4–7^	−0.7 (−11.7 to 10.2)	0.6 (−4.5 to 5.7)	−4.9 (−15.9 to 6.0)	0.5 (−3.9 to 5.0)	4.2 (−12.3 to 20.7)	0.61	0.1 (−7.6 to 7.6)	0.99
Alkaline phosphatase, IU/L								
Intention-to-treat	−1.9 (−9.7 to 5.8)	−3.5 (−6.6 to −0.3)	−4.6 (−9.2 to 0.0)	0.1 (−3.6 to 3.8)	2.7 (−6.1 to 11.4)	0.54	−3.6 (−8.3 to 1.1)	0.13
Intention-to-treat + HEI ^4^	−2.0 (−8.3 to 4.3)	−3.8 (−7.3 to −0.4)	−4.6 (−11.5 to 2.3)	0.4 (−2.9 to 3.8)	2.6 (−6.8 to 12.0)	0.57	−4.3 (−9.2 to 0.7)	0.09
Protocol adherence ^5–7^	0.1 (−7.2 to 7.4)	−4.7 (−9.1 to −0.4)	−7.0 (−14.3 to 0.3)	0.6 (−3.2 to 4.4)	7.1 (−3.9 to 18.1)	0.20	−5.3 (−11.7 to 1.0)	0.10
Protocol adherence + HEI ^4–7^	0.0 (−7.5 to 7.6)	−5.4 (−9.9 to −0.9)	−7.0 (−14.5 to 0.5)	1.1 (−2.8 to 5.1)	7.0 (−4.3 to 18.3)	0.21	−6.5 (−13.3 to 0.2)	0.06
NAFLD fibrosis score								
Intention-to-treat	−0.1 (−0.4 to 0.1)	0.2 (−0.1 to 0.5)	−0.1 (−0.4 to 0.2)	0.4 (0.1 to 0.6)	0.0 (−0.4 to 0.4)	0.98	−0.2 (−0.5 to 0.2)	0.38
Intention-to-treat + HEI ^4^	−0.1 (−0.4 to 0.1)	0.2 (0.0 to 0.5)	−0.1 (−0.4 to 0.2)	0.4 (0.1 to 0.6)	0.0 (−0.4 to 0.4)	0.97	−0.1 (−0.5 to 0.2)	0.49
Protocol adherence ^5–7^	−0.2 (−0.5 to 0.2)	0.3 (0.0 to 0.6)	−0.1 (−0.4 to 0.2)	0.3 (0.0 to 0.6)	0.0 (−0.5 to 0.4)	0.83	0.0 (−0.5 to 0.5)	0.98
Protocol adherence + HEI ^4–7^	−0.2 (−0.5 to 0.2)	0.3 (0.0 to 0.7)	−0.1 (−0.4 to 0.2)	0.3 (0.0 to 0.6)	0.0 (−0.5 to 0.4)	0.84	0.0 (−0.5 to 0.6)	0.87

^1^ Defined as a fasting glucose level ≥100 mg/dL or glycosylated hemoglobin ≥5.7% and/or reported use of glucose-lowering medication, at baseline. ^2^ Mean difference is low-high avocado allotment group. ^3^ From unpaired t-test or ANCOVA model (adjusted for Healthy Eating Index 2015 score at Month 6 and/or intervention adherence), where appropriate. ^4^ Adjusted for Healthy Eating Index 2015 score at Month 6. ^5^ Adjusted for intervention adherence. ^6^ Sample size by intervention group allocation (low avocado allotment/high avocado allotment): 31/35 at month 6. ^7^ Sample size by prediabetes/diabetes status (yes/no) for low avocado allotment: 15/16 and for high avocado allotment: 15/20 at month 6. ALT, Alanine aminotransferase; AST, Aspartate aminotransferase; GGT, gamma-glutamyl transferase; hsCRP, high-sensitivity c-reactive protein; NAFLD, Non-alcoholic fatty liver disease.

**Table 4:** Changes in hepatic function outcomes at 6 months after accounting for change in avocado intake ^1^, per intention-to-treat and protocol adherence analyses.

Outcome	Within-Group Differences		Mean between-Group Difference ^2^ (95% CI)	*p*-Value ^3^
	Low Avocado Allotment	High Avocado Allotment		
	Mean (95% CI)	Mean (95% CI)		
	(*n* = 37)	(*n* = 35)		
hsCRP, mg/L				
Intention-to-treat	−0.1 (−0.7 to 0.5)	−0.3 (−0.9 to 0.4)	0.2 (−0.8 to 1.2)	0.73
Intention-to-treat + HEI ^4^	−0.2 (−0.8 to 0.5)	−0.2 (−0.9 to 0.4)	0.1 (−0.9 to 1.1)	0.91
Protocol adherence ^5,6^	−0.1 (−0.9 to 0.6)	−0.3 (−0.9 to 0.4)	0.2 (−0.9 to 1.2)	0.80
Protocol adherence + HEI ^4–6^	−0.2 (−0.9 to 0.5)	−0.2 (−0.9 to 0.5)	0.0 (−1.1 to 1.1)	0.99
GGT, IU/L				
Intention-to-treat	6.0 (−2.6 to 14.6)	−2.7 (−11.6 to 6.1)	8.7 (−4.5 to 21.9)	0.19
Intention-to-treat + HEI ^4^	5.5 (−3.3 to 14.2)	−2.1 (−11.2 to 6.9)	7.6 (−6.0 to 21.2)	0.27
Protocol adherence ^5,6^	6.7 (−3.0 to 16.4)	−2.3 (−11.3 to 6.8)	8.9 (−5.1 to 23.0)	0.21
Protocol adherence + HEI ^4–6^	6.0 (−3.9 to 15.9)	−1.7 (−10.9 to 7.6)	7.6 (−6.9 to 22.2)	0.30
AST, IU/L				
Intention-to-treat	0.0 (−3.3 to 3.2)	−1.9 (−5.3 to 1.4)	1.9 (−3.2 to 6.9)	0.46
Intention-to-treat + HEI ^4^	−0.1 (−3.4 to 3.3)	−1.9 (−5.4 to 1.6)	1.8 (−3.4 to 7.0)	0.49
Protocol adherence ^5,6^	−0.1 (−3.8 to 3.6)	−1.9 (−5.4 to 1.5)	1.8 (−3.6 to 7.2)	0.50
Protocol adherence + HEI ^4–6^	−0.2 (−4.0 to 3.6)	−1.9 (−5.4 to 1.7)	1.7 (−3.9 to 7.3)	0.54
ALT, IU/L				
Intention-to-treat	0.3 (−4.6 to 5.1)	−2.1 (−7.2 to 2.9)	2.4 (−5.1 to 9.9)	0.53
Intention-to-treat + HEI ^4^	0.2 (−4.8 to 5.1)	−2.0 (−7.2 to 3.1)	2.2 (−5.6 to 10.0)	0.58
Protocol adherence ^5,6^	0.2 (−5.3 to 5.8)	−2.1 (−7.2 to 3.1)	2.3 (−5.7 to 10.3)	0.57
Protocol adherence + HEI ^4–6^	0.1 (−5.5 to 5.8)	−2.0 (−7.2 to 3.3)	2.1 (−6.2 to 10.4)	0.62
Alkaline phosphatase, IU/L				
Intention-to-treat	−2.2 (−5.8 to 1.4)	−2.5 (−6.2 to 1.2)	0.3 (−5.3 to 5.9)	0.91
Intention-to-treat + HEI ^4^	−2.3 (−6.0 to 1.5)	−2.4 (−6.2 to 1.4)	0.2 (−5.6 to 5.9)	0.96
Protocol adherence ^5,6^	−2.6 (−6.7 to 1.5)	−2.5 (−6.3 to 1.3)	−0.1 (−6.0 to 5.8)	0.98
Protocol adherence + HEI ^4–6^	−2.7 (−6.9 to 1.5)	−2.4 (−6.3 to 1.5)	−0.3 (−6.4 to 5.8)	0.92
NAFLD fibrosis score				
Intention-to-treat	0.1 (−0.1 to 0.3)	0.1 (−0.1 to 0.3)	0.0 (−0.3 to 0.3)	0.86
Intention-to-treat + HEI ^4^	0.1 (−0.1 to 0.3)	0.1 (−0.1 to 0.3)	0.0 (−0.3 to 0.4)	0.76
Protocol adherence ^5,6^	0.1 (−0.1 to 0.3)	0.1 (−0.1 to 0.3)	0.0 (−0.3 to 0.4)	0.88
Protocol adherence + HEI ^4–6^	0.1 (−0.1 to 0.4)	0.1 (−0.1 to 0.3)	0.0 (−0.3 to 0.4)	0.78

^1^ Defined as change in participant intake from baseline to Month 6. ^2^ Mean difference is low-high avocado allotment group. ^3^ From ANCOVA model (adjusted for change in avocado intake and/or Healthy Eating Index 2015 score at Month 6 and/or intervention adherence), where appropriate. ^4^ Adjusted for Healthy Eating Index 2015 score at Month 6. ^5^ Adjusted for intervention adherence. ^6^ Sample size by intervention group allocation (low avocado allotment/high avocado allotment): 31/35 at month 6. ALT, Alanine aminotransferase; AST, Aspartate aminotransferase; GGT, gamma-glutamyl transferase; hsCRP, high-sensitivity c-reactive protein; NAFLD, Non-alcoholic fatty liver disease.

**Table 5:** Changes in hepatic function outcomes at 6 months after accounting for change in avocado intake ^1^, stratified by prediabetes status ^2^, per intention-to-treat and protocol adherence analyses.

Outcome	Within-Group Differences				Mean between-Group Difference ^3^ (95% CI)			
	Low Avocado Allotment(*n* = 37)		High Avocado Allotment(*n* = 35)					
	Mean (95% CI)		Mean (95% CI)		Mean (95% CI)	*p*-Value ^4^	Mean (95% CI)	*p*-Value ^4^
	Prediabetes		Prediabetes		Prediabetes		Prediabetes	
	Yes (*n* = 18)	No (*n* = 19)	Yes (*n* = 15)	No (*n* = 20)	Yes		No	
hsCRP, mg/L								
Intention-to-treat	−0.2 (−1.4 to 1.0)	0.0 (−0.6 to 0.6)	−0.3 (−1.6 to 1.1)	−0.3 (−0.9 to 0.3)	0.1 (−1.8 to 1.9)	0.95	0.3 (−0.6 to 1.2)	0.52
Intention-to-treat + HEI ^5^	−0.3 (−1.5 to 0.9)	0.1 (−0.5 to 0.7)	−0.2 (−1.5 to 1.1)	−0.4 (−1.0 to 0.2)	−0.1 (−1.9 to 1.7)	0.92	0.5 (−0.5 to 1.5)	0.31
Protocol adherence ^6–8^	−0.2 (−1.6 to 1.1)	0.0 (−0.7 to 0.7)	−0.2 (−1.6 to 1.1)	−0.3 (−0.9 to 0.3)	0.0 (−2.0 to 2.0)	0.99	0.3 (−0.7 to 1.3)	0.56
Protocol adherence + HEI ^5–8^	−0.3 (−1.7 to 1.0)	0.1 (−0.6 to 0.8)	−0.2 (−1.5 to 1.2)	−0.4 (−1.0 to 0.2)	−0.2 (−2.2 to 1.8)	0.86	0.5 (−0.6 to 1.6)	0.35
GGT, IU/L								
Intention-to-treat	12.2 (−4.3 to 28.6)	−1.9 (−6.5 to 2.6)	−7.5 (−25.7 to 10.6)	2.9 (−1.5 to 7.3)	19.7 (−5.5 to 45.0)	0.12	−4.8 (−11.9 to 2.3)	0.18
Intention-to-treat + HEI ^5^	11.5 (−5.1 to 28.0)	−1.4 (−6.2 to 3.5)	−6.7 (−24.9 to 11.5)	2.4 (−2.3 to 7.0)	18.2 (−7.3 to 43.7)	0.16	−3.7 (−11.4 to 4.0)	0.33
Protocol adherence ^6–8^	13.8 (−5.3 to 32.7)	−2.1 (−7.3 to 3.0)	−6.6 (−25.6 to 12.4)	2.7 (−1.8 to 7.3)	20.3 (−7.2 to 47.8)	0.14	−4.9 (−12.4 to 2.6)	0.20
Protocol adherence + HEI ^5–8^	12.8 (−6.3 to 31.9)	−1.5 (−6.9 to 4.0)	−5.7 (−24.9 to 13.4)	2.2 (−2.5 to 6.9)	18.4 (−9.3 to 46.2)	0.18	−3.7 (−11.8 to 4.4)	0.36
AST, IU/L								
Intention-to-treat	−0.2 (−5.7 to 5.3)	−0.7 (−4.6 to 3.1)	−5.0 (−11.1 to 1.0)	1.2 (−2.6 to 4.9)	4.8 (−3.6 to 13.3)	0.25	−1.9 (−7.8 to 4.0)	0.52
Intention-to-treat + HEI ^5^	−0.5 (−5.9 to 5.0)	0.2 (−3.7 to 4.2)	−4.7 (−10.7 to 1.3)	0.2 (−3.6 to 4.0)	4.2 (−4.2 to 12.6)	0.31	0.0 (−6.3 to 6.2)	0.99
Protocol adherence ^6–8^	−0.2 (−6.5 to 6.2)	−0.8 (−5.1 to 3.5)	−5.1 (−11.4 to 1.3)	1.1 (−2.7 to 4.9)	4.9 (−4.3 to 14.1)	0.28	−1.9 (−8.2 to 4.3)	0.53
Protocol adherence + HEI ^5–8^	−0.6 (−6.9 to 5.7)	0.2 (−4.2 to 4.7)	−4.7 (−11.0 to 1.6)	0.3 (−3.6 to 4.1)	4.1 (−5.1 to 13.3)	0.37	0.0 (−6.6 to 6.6)	0.99
ALT, IU/L								
Intention-to-treat	0.2 (−8.9 to 9.2)	−0.6 (−5.0 to 3.9)	−5.9 (−15.8 to 4.1)	1.5 (−2.8 to 5.8)	6.0 (−7.9 to 19.9)	0.38	−2.1 (−9.0 to 4.8)	0.54
Intention-to-treat + HEI ^5^	−0.2 (−9.3 to 8.9)	0.5 (−4.1 to 5.0)	−5.4 (−15.5 to 4.6)	0.6 (−3.9 to 5.0)	5.2 (−8.8 to 19.3)	0.45	−0.1 (−7.4 to 7.2)	0.98
Protocol adherence ^6–8^	0.2 (−10.2 to 10.7)	−0.7 (−5.6 to 4.3)	−5.9 (−16.3 to 4.5)	1.5 (−2.8 to 5.9)	6.2 (−8.9 to 21.3)	0.41	−2.2 (−9.4 to 5.1)	0.54
Protocol adherence + HEI ^5–8^	−0.2 (−10.7 to 10.3)	0.5 (−4.6 to 5.6)	−5.4 (−16.0 to 5.1)	0.6 (−3.8 to 5.1)	5.2 (−10.1 to 20.5)	0.49	−0.1 (−7.8 to 7.5)	0.97
Alkaline phosphatase, IU/L								
Intention-to-treat	−0.9 (−7.1 to 5.4)	−4.6 (−8.5 to −0.8)	−5.9 (−12.8 to 1.0)	1.2 (−2.5 to 4.9)	5.0 (−4.6 to 14.6)	0.30	−5.8 (−11.8 to 0.1)	0.05
Intention-to-treat + HEI ^5^	−0.9 (−7.3 to 5.5)	−5.3 (−9.3 to −1.3)	−5.9 (−12.9 to 1.2)	1.9 (−2.0 to 5.7)	5.0 (−4.9 to 14.9)	0.31	−7.2 (−13.6 to 0.8)	0.03
Protocol adherence ^6–8^	−1.2 (−8.4 to 6.0)	−5.1 (−9.4 to −0.9)	−5.7 (−12.9 to 1.5)	0.9 (−2.8 to 4.7)	4.5 (−6.0 to 14.9)	0.39	−6.1 (−12.3 to 0.2)	0.06
Protocol adherence + HEI ^5–8^	−1.2 (−8.6 to 6.1)	−5.9 (−10.4 to −1.4)	−5.7 (−13.1 to 1.7)	1.5 (−2.4 to 5.4)	4.4 (−6.3 to 15.2)	0.40	−7.4 (−14.1 to 0.7)	0.03
NAFLD fibrosis score								
Intention-to-treat	−0.1 (−0.4 to 0.1)	0.3 (0.0 to 0.6)	0.1 (−0.4 to 0.2)	0.3 (0.0 to 0.6)	0.0 (−0.4 to 0.4)	0.95	0.0 (−0.4 to 0.5)	0.93
Intention-to-treat + HEI ^5^	−0.1 (−0.4 to 0.1)	0.3 (0.0 to 0.6)	−0.1 (−0.4 to 0.2)	0.3 (0.0 to 0.5)	0.0 (−0.4 to 0.4)	0.95	0.1 (−0.4 to 0.6)	0.74
Protocol adherence ^6–8^	−0.2 (−0.5 to 0.2)	0.3 (0.0 to 0.7)	−0.1 (−0.4 to 0.2)	0.3 (0.0 to 0.6)	0.0 (−0.5 to 0.4)	0.88	0.0 (−0.4 to 0.5)	0.90
Protocol adherence + HEI ^5–8^	−0.2 (−0.5 to 0.2)	0.4 (0.0 to 0.7)	−0.1 (−0.4 to 0.2)	0.3 (0.0 to 0.6)	0.0 (−0.5 to 0.4)	0.88	0.1 (−0.4 to 0.6)	0.73

^1^ Defined as change in participant intake from baseline to Month 6. ^2^ Defined as a fasting glucose level ≥100 mg/dL or glycosylated hemoglobin ≥5.7 % and/or reported use of glucose-lowering medication, at baseline. ^3^ Mean difference is low-high avocado allotment group. ^4^ From ANCOVA model (adjusted for change in avocado intake and/or Healthy Eating Index 2015 score at Month 6 and/or intervention adherence), where appropriate. ^5^ Adjusted for Healthy Eating Index 2015 score at Month 6. ^6^ Adjusted for intervention adherence. ^7^ Sample size by intervention group allocation (low avocado allotment/high avocado allotment): 31/35 at month 6. ^8^ Sample size by prediabetes/diabetes status (yes/no) for low avocado allotment: 15/16 and for high avocado allotment: 15/20 at month 6. ALT, Alanine aminotransferase; AST, Aspartate aminotransferase; GGT, gamma-glutamyl transferase; hsCRP, high-sensitivity c-reactive protein; NAFLD, Non-alcoholic fatty liver disease.

**Table 6:** Between-group differences of multivariable linear regression models assessing 6-month changes in hepatic function markers related to avocado ^1^ allotment, per intention-to-treat and per protocol adherence analyses ^2^.

	Mean between-Group Difference ^3^ (95% CI)							
	Model 1 ^4^	R-Squared	Model 2 ^5^	R-Squared	Model 3 ^6^	R-Squared	Model 4 ^7^	R-Squared
hsCRP, mg/L								
Intention-to-treat ^8^	−0.1 (−0.9 to 0.8)	0.09	0.0 (−0.9 to 0.8)	0.09	0.0 (−0.9 to 0.8)	0.12	−0.1 (−1.0 to 0.8)	0.12
Protocol adherence ^9^	0.0 (−0.9 to 0.9)	0.09	0.0 (−1.0 to 0.9)	0.09	0.0 (−0.9 to 1.0)	0.12	0.0 (−1.0 to 0.9)	0.12
GGT, IU/L								
Intention-to-treat ^8^	−2.4 (−11.7 to 7.0)	0.40	−2.9 (−12.5 to 6.6)	0.41	−2.8 (−12.5 to 6.8)	0.41	−3.0 (−12.9 to 7.0)	0.41
Protocol adherence ^9^	−1.2 (−11.2 to 8.9)	0.44	−1.8 (−12.0 to 8.4)	0.44	−1.7 (−12.0 to 8.6)	0.45	−1.9 (−12.6 to 8.8)	0.45
AST, IU/L								
Intention-to-treat ^8^	0.6 (−3.8 to 4.9)	0.11	1.0 (−3.4 to 5.3)	0.13	1.0 (−3.4 to 5.4)	0.13	1.0 (−3.5 to 5.6)	0.13
Protocol adherence ^9^	0.6 (−4.2 to 5.3)	0.11	1.0 (−3.8 to 5.8)	0.14	1.0 (−3.9 to 5.8)	0.14	1.1 (−4.0 to 6.1)	0.14
ALT, IU/L								
Intention-to-treat ^8^	0.2 (−6.3 to 6.7)	0.09	0.4 (−6.2 to 7.0)	0.09	0.3 (−6.4 to 6.9)	0.10	0.2 (−6.7 to 7.1)	0.10
Protocol adherence ^9^	0.3 (−6.9 to 7.4)	0.09	0.4 (−6.9 to 7.8)	0.09	0.3 (−7.1 to 7.6)	0.10	0.1 (−7.5 to 7.8)	0.10
Alkaline phosphatase, IU/L								
Intention-to-treat ^8^	−2.1 (−6.6 to 2.4)	0.23	−2.3 (−6.9 to 2.2)	0.23	−2.3 (−6.9 to 2.3)	0.23	−2.1 (−6.8 to 2.6)	0.24
Protocol adherence ^9^	−2.4 (−7.2 to 2.5)	0.24	−2.6 (−7.6 to 2.3)	0.24	−2.6 (−7.6 to 2.4)	0.24	−2.4 (−7.6 to 2.8)	0.25
NAFLD fibrosis score								
Intention-to-treat ^8^	−0.2 (−0.4 to 0.1)	0.13	−0.2 (−0.4 to 0.1)	0.14	−0.2 (−0.4 to 0.1)	0.16	−0.1 (−0.4 to 0.1)	0.17
Protocol adherence ^9^	−0.2 (−0.5 to 0.1)	0.13	−0.2 (−0.5 to 0.1)	0.14	−0.2 (−0.5 to 0.1)	0.17	−0.1 (−0.4 to 0.2)	0.18

^1^ Allocated avocado group in the trial; thus, intervention status was a covariate in the model. ^2^ All models had *p*-value >0.05 unless otherwise stated. ^3^ Mean difference is low-high avocado allotment group. ^4^ Model 1 adjusted for: age (years); MVPA (minutes/week); total energy intake (kcals/day); alcohol (grams/day). ^5^ Model 2 adjusted for: variables in Model 1 and change in fruit and vegetable intake (servings/day). ^6^ Model 3 adjusted for: variables in Model 2 and change in waist circumference (cm). ^7^ Model 4 adjusted for: variables in Model 3 and HEI 2015 score at Month 6. ^8^ *n* = 72. ^9^ *n* = 66. ALT, Alanine aminotransferase; AST, Aspartate aminotransferase; GGT, gamma-glutamyl transferase; hsCRP, high-sensitivity c-reactive protein; NAFLD, Non-alcoholic fatty liver disease.

**Table 7:** Between-group differences of multivariable linear regression models assessing 6-month changes in hepatic function markers related to avocado ^1^ allotment, per intention-to-treat and per protocol adherence analyses ^2^, stratified by prediabetes status ^3^.

	Presence of Prediabetes							
	Mean between-Group Difference ^4^ (95% CI)							
	Model 1 ^5^	R-Squared	Model 2 ^6^	R-Squared	Model 3 ^7^	R-Squared	Model 4 ^8^	R-Squared
hsCRP, mg/L								
Intention-to-treat ^9^	−0.4 (−2.1 to 1.2)	0.26	−0.5 (−2.3 to 1.2)	0.26	−0.3 (−2.1 to 1.5)	0.29	−0.5 (−2.3 to 1.4)	0.31
Protocol adherence ^10^	−0.5 (−2.3 to 1.4)	0.26	−0.6 (−2.6 to 1.4)	0.27	−0.4 (−2.4 to 1.7)	0.30	−0.6 (−2.7 to 1.6)	0.32
GGT, IU/L								
Intention-to-treat ^9^	5.4 (−6.7 to 17.5)	0.81	5.3 (−7.7 to 18.2)	0.81	4.4 (−9.1 to 17.9)	0.82	4.4 (−9.1 to 17.9)	0.82
Protocol adherence ^10^	8.5 (−3.1 to 20.1)	0.87	8.4 (−4.1 to 20.8)	0.87	7.3 (−5.7 to 20.2)	0.88	6.1 (−7.5 to 19.7)	0.88
AST, IU/L								
Intention-to-treat ^9^	3.9 (−3.2 to 10.9)	0.37	4.9 (−2.6 to 12.3)	0.39	5.0 (−2.8 to 12.8)	0.39	4.5 (−3.6 to 12.7)	0.40
Protocol adherence ^10^	3.8 (−4.3 to 11.9)	0.39	4.9 (−3.6 to 13.4)	0.41	5.1 (−3.9 to 14.0)	0.41	4.3 (−5.1 to 13.7)	0.42
ALT, IU/L								
Intention-to-treat ^9^	5.5 (−6.1 to 17.1)	0.36	6.5 (−5.8 to 18.8)	0.37	6.9 (−6.0 to 19.9)	0.37	6.9 (−6.7 to 20.4)	0.37
Protocol adherence ^10^	5.9 (−7.3 to 19.1)	0.38	7.1 (−6.9 to 21.1)	0.39	7.6 (−7.2 to 22.4)	0.39	7.2 (−8.5 to 22.9)	0.40
Alkaline phosphatase, IU/L								
Intention-to-treat ^9^	1.8 (−6.2 to 9.9)	0.38	2.6 (−5.9 to 11.1)	0.39	2.9 (−6.0 to 11.9)	0.39	3.9 (−5.4 to 13.1)	0.42
Protocol adherence ^10^	1.7 (−7.5 to 10.8)	0.40	2.5 (−7.3 to 12.2)	0.41	2.8 (−7.5 to 13.1)	0.41	3.9 (−6.8 to 14.6)	0.43
NAFLD fibrosis score								
Intention-to-treat ^9^	0.0 (−0.4 to 0.3)	0.22	−0.1 (−0.4 to 0.3)	0.22	−0.1 (−0.5 to 0.4)	0.22	0.0 (−0.5 to 0.4)	0.22
Protocol adherence ^10^	−0.2 (−0.6 to 0.2)	0.28	−0.2 (−0.6 to 0.3)	0.28	−0.2 (−0.6 to 0.3)	0.28	−0.2 (−0.7 to 0.3)	0.28
	**Absence of Prediabetes**							
	**Mean between-Group Difference ^4^ (95% CI)**							
	**Model 1 ^5^**	**R-Squared**	**Model 2 ^6^**	**R-Squared**	**Model 3 ^7^**	**R-Squared**	**Model 4 ^8^**	**R-Squared**
hsCRP, mg/L								
Intention-to-treat ^9^	0.0 (−0.7 to 0.7)	0.28	0.0 (−0.9 to 0.8)	0.09	0.0 (−0.9 to 0.8)	0.12	−0.1 (−1.0 to 0.8)	0.12
Protocol adherence ^10^	0.0 (−0.8 to 0.8)	0.28	0.0 (−0.8 to 0.8)	0.30	0.0 (−0.8 to 0.8)	0.30	0.0 (−1.0 to 0.9)	0.30
GGT, IU/L								
Intention-to-treat ^9^	−3.8 (−9.8 to 2.3)	0.15	−3.9 (−9.8 to 2.0)	0.22	−4.5 (−10.3 to 1.2)	0.29	−4.5 (−11.0 to 1.9)	0.29
Protocol adherence ^10^	−3.9 (−10.4 to 2.7)	0.15	−4.0 (−10.4 to 2.4)	0.22	−4.5 (−10.8 to 1.7)	0.29	−4.4 (−11.5 to 2.6)	0.29
AST, IU/L								
Intention-to-treat ^9^	1.6 (−6.9 to 3.7)	0.03	−1.5 (−6.8 to 3.8)	0.07	−1.6 (−7.0 to 3.8)	0.07	0.0 (−5.8 to 5.9)	0.13
Protocol adherence ^10^	−1.6 (−7.3 to 4.2)	0.04	−1.5 (−7.2 to 4.2)	0.07	−1.6 (−7.4 to 4.3)	0.08	0.3 (−6.1 to 6.6)	0.14
ALT, IU/L								
Intention-to-treat ^9^	−2.2 (−8.2 to 3.9)	0.07	−2.2 (−8.3 to 4.0)	0.07	−2.1 (−8.3 to 4.2)	0.07	−0.4 (−7.3 to 6.5)	0.12
Protocol adherence ^10^	−2.3 (−8.8 to 4.2)	0.09	−2.3 (−8.9 to 4.4)	0.09	−2.2 (−8.9 to 4.6)	0.09	−0.2 (−7.6 to 7.2)	0.14
Alkaline phosphatase, IU/L								
Intention-to-treat ^9^	−4.0 (−9.2 to 1.2)	0.16	−4.1 (−9.1 to 0.9)	0.24	−4.6 (−9.5 to 0.3) ^11^	0.30	−5.0 (−10.5 to 0.4)	0.31
Protocol adherence ^10^	−4.5 (−10.0 to 1.1)	0.17	−4.6 (−10.0 to 0.8)	0.25	−5.0 (−10.3 to 0.3) ^11^	0.31	−5.6 (−11.5 to 0.4) ^11^	0.32
NAFLD fibrosis score								
Intention-to-treat ^9^	−0.3 (−0.6 to 0.1)	0.16	−0.2 (−0.6 to 0.1)	0.19	−0.2 (−0.6 to 0.2)	0.20	−0.1 (−0.5 to 0.3)	0.24
Protocol adherence ^10^	−0.2 (−0.6 to 0.2)	0.17	−0.2 (−0.6 to 0.2)	0.20	−0.2 (−0.6 to 0.2)	0.20	−0.1 (−0.6 to 0.4)	0.24

^1^ Allocated avocado group in the trial; thus, intervention status was a covariate in the model. ^2^ All models had *p*-value > 0.05 unless otherwise stated. ^3^ Defined as a fasting glucose level ≥100 mg/dL or glycosylated hemoglobin ≥5.7% and/or reported use of glucose-lowering medication, at baseline. ^4^ Mean difference is low-high avocado allotment group. ^5^ Model 1 adjusted for: age (years); MVPA (minutes/week); total energy intake (kcals/day); alcohol (grams/day). ^6^ Model 2 adjusted for: variables in Model 1 and change in fruit and vegetable intake (servings/day). ^7^ Model 3 adjusted for: variables in Model 2 and change in waist circumference (cm). ^8^ Model 4 adjusted for: variables in Model 3 and HEI 2015 score at Month 6. ^9^ Sample size by intervention group allocation (low avocado allotment/high avocado allotment): 37/35 at baseline; where by prediabetes/diabetes status (yes/no) for low avocado allotment: 18/19 and for high avocado allotment: 15/20. ^10^ Sample size by intervention group allocation (low avocado allotment/high avocado allotment): 31/35 at month 6; where by prediabetes/diabetes status (yes/no) for low avocado allotment: 15/16 and for high avocado allotment: 15/20. ^11^ *p*-value = 0.06. ALT, Alanine aminotransferase; AST, Aspartate aminotransferase; GGT, gamma-glutamyl transferase; hsCRP, high-sensitivity c-reactive protein; NAFLD, Non-alcoholic fatty liver disease.

## Data Availability

Data described in the manuscript, code book, and analytic code will be made available upon re-quest pending application and approval. Proposals should be directed to malli-son@health.ucsd.edu.
